# TOP2A Promotes Lung Adenocarcinoma Cells' Malignant Progression and Predicts Poor Prognosis in Lung Adenocarcinoma

**DOI:** 10.7150/jca.41415

**Published:** 2020-02-10

**Authors:** Fan Kou, Houfang Sun, Lei Wu, Baihui Li, Bailu Zhang, Xuezhou Wang, Lili Yang

**Affiliations:** 1 Department of Immunology, Tianjin Medical University Cancer Institute and Hospital, Tianjin, China.; 2 National Clinical Research Center for Cancer, Tianjin, China.; 3 Key Laboratory of Cancer Immunology and Biotherapy, Tianjin, China.; 4 Key Laboratory of Cancer Prevention and Therapy, Tianjin, China.; 5 Tianjin's Clinical Research Center for Cancer, Tianjin, China.

**Keywords:** topoisomerase IIA, lung cancer, p53 pathway, tumorigenesis

## Abstract

**Background**: Topoisomerase IIA (TOP2A) gene encodes DNA topoisomerase enzyme and has been reported that TOP2A is broadly expressed in many types of cancers. Our study aims to investigate the prognostic effect of TOP2A on lung adenocarcinoma (LUAD) and the potential molecular mechanism of TOP2A to tumorigenesis.

**Methods**: Bioinformatical analysis, real-time PCR and Western blot were applied to explore the expression level of TOP2A. Kaplan-Meier survival analysis was used to evaluate the effect of TOP2A on patients' prognosis. Cell proliferation, migration and invasion ability were examined by colony-formation, Cell Counting Kit-8 (CCK8) assay, wound healing assay and transwell invasion assay, respectively.

**Results**: We firstly investigated differentially expressed genes in lung adenocarcinoma and normal tissues of GEO (tumor = 666, normal = 184) and TCGA (tumor = 517, normal = 59) and these data showed that TOP2A is broadly expressed in LUAD and the expression level of TOP2A is associated with poor prognosis, which indicated that TOP2A is an upregulated prognostic related gene in LUAD. Then we identified that the expression level of TOP2A was upregulated in both surgically removed lung cancer tissues and lung cancer cell lines. Knockdown of TOP2A in A549 and GLC82 cells inhibited cell proliferation, migration and invasion. Inhibition of TOP2A reduced the expression levels of CCNB1 and CCNB2, which indicated that TOP2A targeting CCNB1 and CCNB2 promotes GLC82 and A549 cells proliferation and metastasis.

**Conclusions**: Our study revealed an important role of TOP2A in LUAD, and may provide a potential prognostic indicator and target for cancer therapy.

## Introduction

Lung cancer is the most common cause of cancer-related mortality in the world and composed of small-cell lung cancer and non-small-cell lung cancer (NSCLC) 1. NSCLC accounts for more than 80% of the diagnosed lung cancer 2, 3. Although improved methods of detection and management of patients with lung cancer, the 5-year relative survival rate of lung cancer is lower than 18% 1, 4, which shows that finding novel therapeutic target has been a rising challenge against lung cancer.

DNA topoisomerase family has two subfamilies: type I topoisomerase inducing DNA single strand breaks and type II topoisomerase inducing double strand breaks in DNA 5. According to different amino acid sequence structures and/or relationships, DNA topoisomerase further divides into: IA, IB, IC, IIA and IIB 6. Type IIA topoisomerase (TOP2A) modulates the topologic structure through relaxing positive and negative DNA supercoils and resolves chromosome condensation and chromatid separation during DNA replication and transcription 5, 7, 8. Additionally, DNA topoisomerase IIA massively accumulates in rapidly dividing cells and TOP2A-specific targets are related to proliferation 7. Accumulating researches have shown that TOP2A is overexpressed in many types of tumors, such as pancreatic cancer, breast cancer, prostate cancer, colon cancer, gastroesophageal and esophageal cancer, hepatoblastoma and malignant peripheral nerve sheath tumor 9-12. Also, TOP2A as a prognostic factor and a driver gene is associated with survival of breast cancer and prostate cancer 13, 14. Inhibition TOP2A results in alterations of signaling and the following pathways: overexpression of TOP2A actives β-catenin pathway in pancreatic cancer 12; downregulation of TOP2A inhibits the activity of EPK and AKT in colon cancer 15. Whereas, the functional role of TOP2A and involving pathways in lung adenocarcinoma (LUAD) are not clear. Based on previous reports, TOP2A might be involved in physiological functions in LUAD.

This study was conducted to investigate TOP2A expression level, prognostic value in lung adenocarcinoma and the mechanism of proliferation and invasion. We report that TOP2A expression is upregulated in LUAD and TOP2A targeting CCNB1 and CCNB2 promotes GLC82 and A549 cells proliferation and metastasis.

## Materials and Methods

### GEO and TCGA data set

The original datasets containing LUAD and normal tissues were obtained from the GEO database (https:// www.ncbi.nlm.nih.gov/geo) and the TCGA data portal (http://tcga-data.nci.nih.gov/tcga/). We downloaded gene expression profiles of GSE10072, GSE43458, GSE83213 from the GEO database. The three GEO datasets are all obtained from mRNA expression profiling of LUAD and matched non- tumor tissues and the total sample amounts are greater than 50. The microarray data of GSE10072, GSE43458 and GSE83213 were separately based on GPL96 (Affymetrix Human Genome U133A Array, Affymetrix, Inc., Santa Clara, CA, USA), GPL6244 (Affymetrix Human Gene 1.0 ST Array, Affymetrix, Inc., Santa Clara, CA, USA) and GPL10558 (Illumina HumanHT-12 V4.0 expression beadchip, Illumina Inc., San Diego, CA, USA). We also analyzed prognoScan database (http://gibk21.bse.kyutech.ac.jp/PrognoScan/index.html) 16 and downloaded the microarray GSE116969 for validating the expression of TOP2A. The original expression data were performed background correction and quantile normalization by robust multi-array average algorithm (RMA) approach in R software 17. Then limma package was applied to identify DEGs between LUAD and normal tissues in each GEO datasets, respectively. Significantly DEGs was identified using a cut-off criterion of |log2FC| > 1.0 and *P* value <0.05 18. The level 3 RNA-seq data (including 517 LUAD and 59 normal tissues) was downloaded from TCGA, Bioconductor package edge R was employed for the screening of DEGs with the same criterion. Finally, vennDiagram package was utilized to find common DEGs among the four datasets.

In the present study, STRING online database (http://string-db.org/) and Cytoscape software (version 3.7.2, http://www.cytoscape.org/) were employed for exploring the interactive relationships of the common DEGs and constructing a PPI network. Cytoscape plug-in cytoHubba was applied to select the hub genes in the PPI network and degree score ≥10 was deemed as the standard. Pathway enrichment analysis was conducted using DAVID online database (version 6.8, https://david.ncifcrf.gov), and *P* < 0.05 was chosen as the threshold to identify the statistically meaningful pathway 19.

### Human lung adenocarcinoma tissues and Immunohistochemistry

All lung adenocarcinoma samples were provided from Tianjin Medical University Cancer Hospital. Eight pairs of lung adenocarcinoma and adjacent normal tissues and eighty-four lung adenocarcinoma tissues were obtained from patients. Patients did not receive neoadjuvant therapy or chemotherapy before surgery. The samples were evaluated and identified by a pathologist. All LUAD patients were followed up until July 2018. The overall survival (OS) and recurrence-free survival (RFS) were the period after the surgery to death or to the time of last follow-up and the period from surgery to recurrence. Patients lost to follow-up were not included in the analysis.

Paraffin-embedded sections were baked at 70°C overnight, then deparaffinized and rehydrated with xylene and ethanol. The sections were soaked in citrate buffer in a microwave for 15 min. After recovering room temperature, the sections were treated with 0.3% hydrogen peroxide for 20 min to quench the endogenous peroxidase activity, and then incubated with a rabbit monoclonal anti-TOP2A antibody (1:400) overnight at 4°C. After washing with phosphate buffered saline (PBS), sections were incubated with secondary antibody. After washing with PBS, added a secondary antibody of undiluted horseradish peroxidase (HRP)-conjugated goat anti-rabbit to the sections at 37°C for 30 min. The pathological sections were immersed in 3, 3'-Diaminobenzidine (DAB) for 3 minutes, counterstained with 10% Mayer's hematoxylin, dehydrated. Two pathologists, unknown to the clinical result, scored the results independently. According to the staining intensities from light to general and dark brown, the expression levels were classified in three degrees: weakly, moderately, and strongly positive respectively. Based on the cut-off value of ROS curve analysis, A TOP2A expression score < 9.0 was described as low expression group while ≥ 9.0 as high expression group.

### Cell culture and transfection

All cell lines were purchased from ATCC. GLC82, A549 and LTEP-A2 were cultured in RPMI-1640 medium supplemented with 10% fetal bovine serum (FBS). BEAS-2B was cultured in BEAS-2B specialized medium supplemented with 10% fetal bovine serum. All cells were incubated in a humidified incubator containing 5% CO2 at 37°C. The TOP2A siRNA and control molecules were transfected with Lipofectamine 3000 according to the manufacturer's manual when cells reached 50% confluence. After transfection, GLC82 and A549 cells were cultured in RPMI medium containing 10% FBS at 37 °C with 5% CO_2_, which were incubated every 48 h. Cell transfection efficiencies and target gene expressions were detected by quantitative real-time PCR (qRT-PCR) and Western blot. The sequences of TOP2A siRNA were as follows: siRNA1: sense 5'GGUCAGAAGAGCAUAUGAUTT3', antisense 5'AUCAUAUGCUCUUCUGACCTT3'; siRNA2: sense 5'GACCAACCUUCAACUAUCUTT3', antisense 5'AGAUAGUUGAAGGUUGGUCTT3'.

### Real-time PCR and Western blot

Logarithmic growth phase cells (1-5 × 10^6^) were collected and the supernatant was removed and washed 2 times using PBS. RNA was extracted via adding 1 ml of Trizol solution and 0.2 ml of chloroform processed according to the manufacturer's protocol. RNA quality and purity were analyzed on UV absorption. The *A*_260_/*A*_280_ ratio of RNA was 1.8 to 2.0 and the cDNA was stored at -20 °C. qRT-PCR was performed with TOP2A primer sequences and beta-actin as internal reference using SYBR Premix EX Taq and the comparative Ct method (2^-ΔΔCt^) method was used to analyze the relative expression. The sequences of primers were as follows: TOP2A: forward primer: 5'CTAGTTAATGCTGCGGACAACA3', reverse primer: 5'CATTTCGACCACCTGTCACTT3'; B-actin: forward primer: 5'TGGCACCCAGCAC AATGAA3', reverse primer: 5'CTAAGTCATAGTCCGCCTAGAAGC3'.

Protein lysates in radioimmunoprecipitation assay (RIPA) buffer combined with cocktail buffer for 30 minutes. Protein extraction and concentration measurement were determined according to the manufacturer's instructions. Protein lysates were isolated by SDS-PAGE, then moved to a PVDF membrane. The membranes were immunoblotted with anti-cyclinB1 (ab32053, abcam), anti-cyclinB2 (ab185622, abcam), anti-chk1(ab79758, abcam), anti-MDM2 (ab170880, abcam), anti-p53, anti-GAPDH and anti-B-actin antibodies at 4°C on a rocking platform shaker overnight. Anti-p53, anti-GAPDH and anti-B-actin antibodies were purchased from Cell Signaling Technology. The membranes were washed with TBST, then incubated Rabbit Antibody for 1 hours at room temperature and washed with TBST.

### Colony-formation and CCK8 assay

Cell proliferation was determined by colony formation. GLC82 and A549 cells transfected with TOP2A siRNA/control were seeded into 12-well plates (100/well), incubating for 10-12 days at 37°C in 5% humidified CO_2_. Colonies were stained with Crystal violet for 15-30 minutes and counted. For CCK8 assay, the cells were washed, collected, and added 100 ul cell suspension (2×10^4^ cells/mL) in each well in 96-well plates after 48 hours of incubating GLC82 and A549 cells transfected with TOP2A siRNA/control. CCK8 (10 ul per well) solution was added 1-4hours before incubation was completed and OD value (a spectrophotometer at an absorbance of 450 nm) was measured in 0, 24, 48, 72 hours. Cells without transfection measured cell proliferation with similar method, seen as blank groups. Cell proliferation was evaluated that the OD value of cells with transfection minus the blank groups.

### Wound healing assay and transwell assay

GLC82 and A549 cells migration was measured by wound-healing assay. The cells (5 × 10^5^ cells/well) transfected with TOP2A siRNA/control were placed in 6-well plates with serum-free medium. After the cell confluence reached 90%, a scratch was performed with a 20ul pipette tip across the cell monolayer and captured at 0, 12, 24 hours. The migration distance was quantified by measuring the distance between the wound edges.

Invasion was examined in 24 transwell chambers using inserts with 8-μm pore membrane. After 6 hours of adding Matrigel to the upper champers, GLC82 and A549 cells were placed in the upper chamber (5 × 10^4^cells/well) in RPMI-1640 medium without FBS. Medium with 10%FBS was seeded into the lower chamber. After 18 h of incubation, the upper chambers cells removed to the lower chambers through Matrigel, stained with Crystal violet for 15-30 minutes, photographed and visually counted in 3 random fields.

### Statistical analysis

Statistical analysis was performed using GraphPad Prism 8 and expressed as mean ± SD. One-way ANOVA tests were applied to multiple comparisons. Kaplan-Meier survival curve was performed to analyze survival data. Significant differences between the groups are indicated by NS for no significant difference, * for *P* < 0.05, ** for *P* < 0.01, *** for *P* < 0.001, *** for *P* < 0.0001.

## Results

### TOP2A is identified as an upregulated prognostic related gene in lung adenocarcinoma tissues

**Figure 1:**
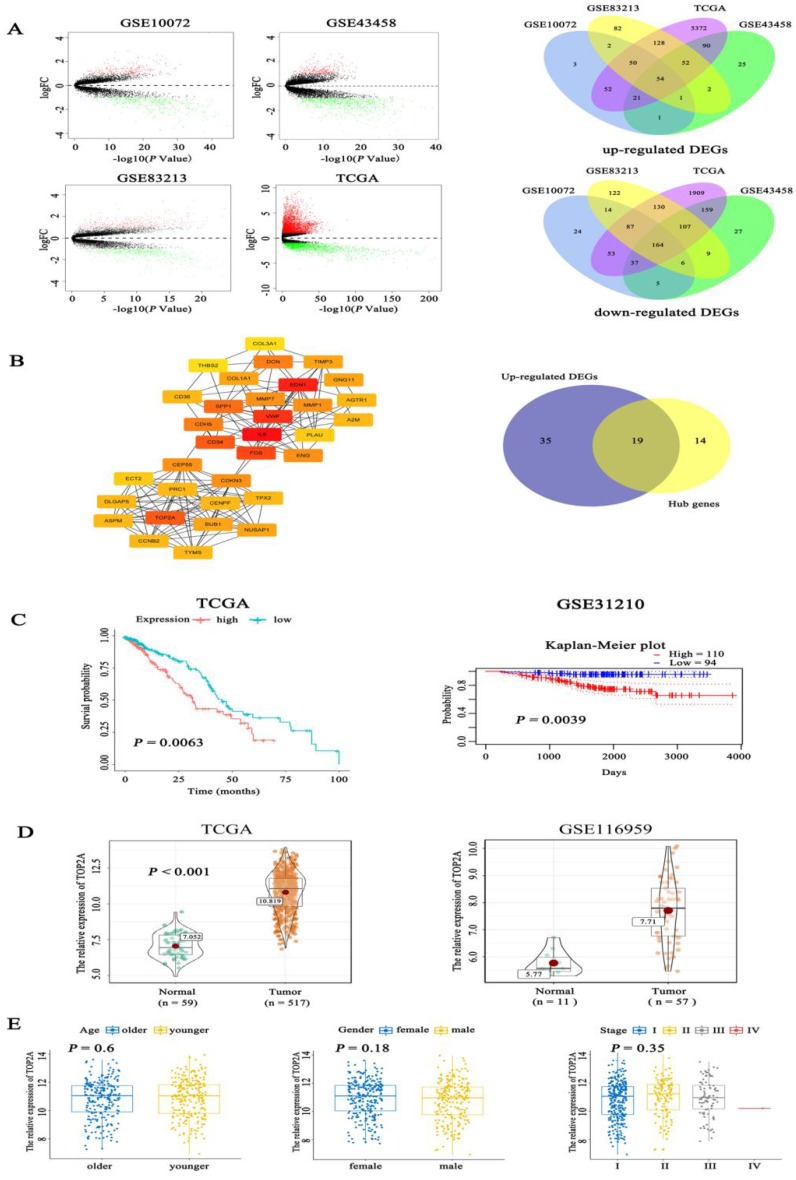
Identification of prognostic related genes in lung adenocarcinoma. A. By analysis of GEO and TCGA datasets, volcano plots showed significantly different expressed genes (DEGs) between LUAD tissues and normal controls. Venn digram further revealed the number of common up-regulated DEGs and down-regulated DEGs among the four datasets. B. Fifty-four hub genes screened out from Cytosacpe cytoHubba plug-in and 19 up-regulated hub genes intersected by R software. C. Kaplan-Meier survival analysis was performed to analyze 504 LUAD patients in TCGA and found that the expression level of TOP2A (*P* = 0.0063) was inversely associated with the prognosis of LUAD patients and the result was validated in GSE31210 by pronoScan online database (*P* = 0.0039). D. We identified the expression of TOP2A in 517 LUAD cases and 59 normal tissues in TCGA, which showed TOP2A was significantly highly expressed in lung adenocarcinoma. The result was validated by GSE116959 (tumor = 57, normal = 11), which found TOP2A was overexpressed in LUAD (*P* = 0.0039). E. The boxplots showed the relationship between clinicopathological parameters and TOP2A expression, which revealed that the expression of TOP2A was not associated with the age (*P* = 0.6), gender (*P* = 0.18), and TNM stage (*P* = 0.35) of patients.

Firstly, we examined the expression level of TOP2A in lung adenocarcinoma and normal tissues using bioinformatics analysis of GSE10072 (tumor = 58, normal =49), GSE43458 (tumor =80, normal = 30), GSE83213 (tumor = 11, normal =46) from GEO and TCGA (tumor = 517, normal =59). Based on a cut-off criterion as |log2FC| > 1.0 and *P* value <0.05, a total of 218 (54 up-regulated and 164 down-regulated) common differentially expressed genes (DEGs) were found in these datasets (Fig. 1A). To further screen out the pivotal genes involved in generation and progression of LUAD, these 218 DEGs were analyzed in STRING database and a PPI network was conducted by Cytoscape software (Fig. S1A), afterwards 33 hub genes (degree > 10, the color shade of node reflects degree) were selected through cytoHubba plug-in and 19 up-regulated hub genes were obtained (Fig. 1B). The detailed name and degree of the 19 genes were displayed in Table S1. To explore the relationship between the 19 genes and the prognosis of LUAD patients, we did survival analysis of these genes with overall survival (OS) information of 504 patients in TCGA. The Kaplan-Meier curves (Fig. 1C) showed that expression level of TOP2A was inversely associated with the prognosis of LUAD patients (*P* = 0.0063). Among the 19 hub genes, the expression level of TOP2A was inversely associated with the prognosis of LUAD patients (P = 0.0063). However, the expression levels of the remaining 18 genes were not significantly related to the prognosis of patients (all *P* > 0.01) (Fig. S1B). The Kaplan-Meier plot of TOP2A in GSE31210 acquiring from prognoScan database further confirmed above conclusion (P = 0.0039). Nevertheless, the role of TOP2A in lung adenocarcinoma remains poorly understood, whether TOP2A is a potentially significant prognostic indicator still needs to be investigated. Besides, we determined the expression of TOP2A in 517 LUAD cases and 59 normal tissues in TCGA, which showed TOP2A was significantly highly expressed in lung adenocarcinoma. The consequence was validated in the latested microarray dataset GSE116959 (tumor = 57, non-tumor =11), which was consistent with previous result (Fig. 1D). To further investigate whether different age, gender and TNM stage have an impact on TOP2A expression, clinicopathological parameters were analyzed to reveal that the expression level of TOP2A was not apparently connected with the age, gender, and TNM stage of patients (all *P* > 0.05) (Fig. 1E).

### TOP2A is expressed in lung adenocarcinoma tissues and related to poor prognosis

**Figure 2:**
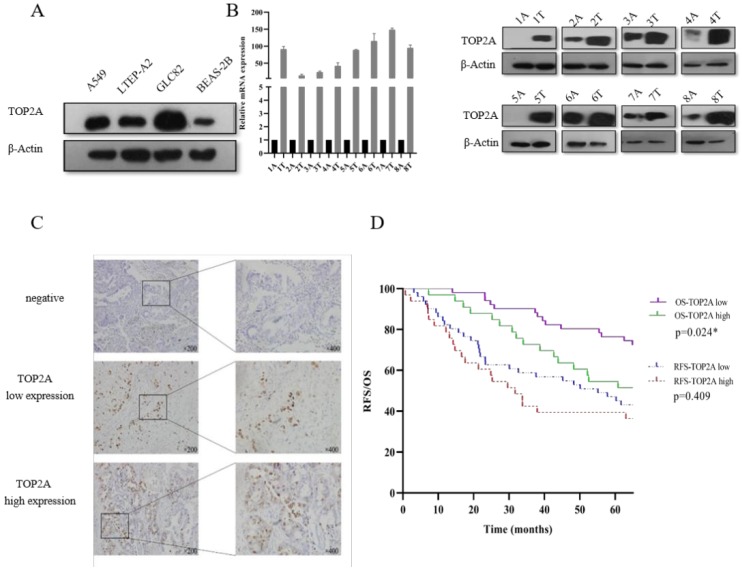
Validation of TOP2A expression in lung adenocarcinoma cell lines and tissues. A. TOP2A expression was performed with Western blot in lung adenocarcinoma cells (A549, LTEP-A2 and GLC82) and normal human bronchial epithelium cell (BEAS-2B). The expression levels of GLC82 and A549 were higher than LTEP-A2 and BEAS-2B; B. TOP2A expression was detected in protein and mRNA levels in 8 lung adenocarcinoma tissues and paired adjacent normal tissues. TOP2A expression was higher in tumor than adjacent normal tissues; C. Representative immunohistochemical imagines for TOP2A in 84 patients with primary lung adenocarcinoma. Original magnification:200× and 400×; D. Correlation analysis between the expression of TOP2A (low and high expression) and recurrence-free survival, overall survival in AD patients by Kaplan-Meier survival curve estimation. TOP2A high expression is related to prognosis. *<0.05.

In the present study, we focused on the TOP2A to explore the mechanism to the tumorigenesis of LUAD. Firstly, we explored the expression level of TOP2A in human lung adenocarcinoma cells (A549, LTEP-A2 and GLC82) and normal human bronchial epithelium cell (BEAS-2B). We found that the expression level of TOP2A was upregulated in A549, LTEP-A2 and GLC82 compared with BEAS-2B (Fig. 2A). To evaluate the expression level of TOP2A in lung adenocarcinoma, a total of 16 matched clinical lung adenocarcinoma tissues and adjacent normal tissues were examined for TOP2A mRNA and protein expression with real-time PCR and Western blot, respectively. These results showed that the mRNA and protein expression levels were significantly upregulated in tumor tissues in contrast to adjacent normal tissues (Fig. 2B).

To further confirm these observations, eighty-four patients with primary adenocarcinoma identified by pathological diagnosis were stained for TOP2A (Figure 2C). To explore the association between TOP2A expression and clinical prognosis, we evaluated the expression level of TOP2A in LUAD patient specimens. Of the 84 LUAD patients, thirty-nine (46.4%) patients were grouped to a TOP2A high expression and forty-five (53.6%) had a TOP2A low expression (including negative stain). A TOP2A expression score < 9.0 was considered as low expression and ≥ 9.0 was high expression. Correlation between TOP2A level and clinicopathologic characteristics of LUAD patients was shown in Table 1. However, the statistical analyses indicated that the expression level of TOP2A was not significantly correlated with TNM stages, smoking index, age and sex. This data showed that TOP2A was an independent prognostic factor.

Considering clinical characteristics, recurrence and death are major reference value in LUAD patient management and are related with prognosis and quality of life. Therefore, we investigated recurrence-free survival (RFS) and overall survival (OS) times between the two TOP2A expression level groups; the median RFS and OS in the high TOP2A expression were 30.062 and 66.957 months, respectively (Table 2), whereas that in the low TOP2A expression were 60.222 and 67.154 months, respectively. Kaplan-Meier survival analysis indicated that OS for LUAD patients with low TOP2A expression was significantly better than patients with high TOP2A expression (*P* = 0.024) (Fig. 2D). RFS for high TOP2A expression patients was worse than patients with low TOP2A expression, but there was no significant (*P* = 0.409). Also, on Cox proportional Hazards regression analysis, TOP2A expression in lung adenocarcinoma was an important risk factor for overall survival (hazard ratio (HR), 2.903; 95% confidence interval (CI), 1.019-4.299; *P* = 0.044) between low and high expression of TOP2A groups. Thus, we hypothesize that TOP2A might promote the growth and metastasis of cancer cells.

### TOP2A promotes lung adenocarcinoma cells proliferation, invasion and migration

**Figure 3:**
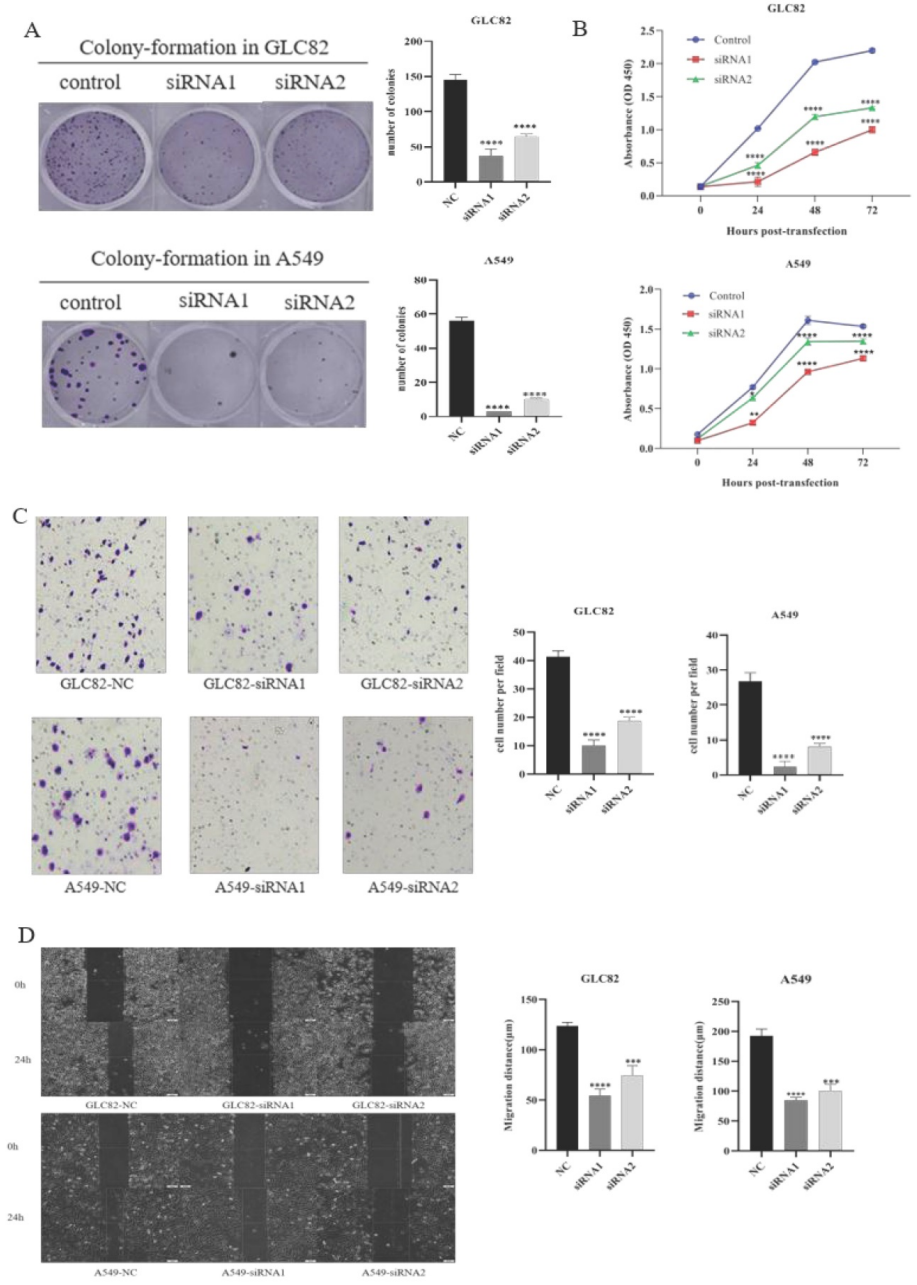
Effect of knockdown TOP2A on the proliferation, invasion and migration of the lung adenocarcinoma cells. A. Owing to GLC82 and A549 with higher TOP2A expression, TOP2A knockdown was performed in GLC82 and A549 and detected by real-time PCR and Western blot. The mRNA and protein levels of GLC82 and A549 transfected with siRNA were lower than control. GLC82 and A549 cells were transfected with control or siRNA for 48h (B, C and D). B. A colony-formation assay was measured to the growth of cells. GLC82 and A549 cells were seeded into 12-well plates(100/well), incubating for ten days. Colonies were counted. C. CCK8 assay was performed to determine the proliferation of GLC82 and A549 cells. Cells were seeded into 96-well plates(2000/well) and OD value was measured in 0,24,48,72 hours. (B, C) Knocking down of TOP2A inhibits the proliferation of lung adenocarcinoma cells. D. Invasion was determined by transwell assay. 50,000 cells per upper chamber, without FBS, were incubated for 18 hours. Knockdown of TOP2A inhibits the invasion of lung adenocarcinoma cells. Representative imagines were captured after 18 hours (magnification, ×100). E. Migration was performed with wound healing assay. 5×10^5^ cells transfected with control or siRNA were added to 6-well plates with serum-free medium. When the cell confluence reached 90%, a scratch was performed with a 20ul pipette tip and captured at 0,12,24 hours. Top2A knockdown inhibits the migration of lung adenocarcinoma cells. The data represent the mean ± S.D. from three independent experiments. *P < 0.05, **P < 0.01, ***P < 0.001, ***P < 0.0001.

Then we investigate the potential function of TOP2A in tumor progression and metastasis. Firstly, we successfully knocked down TOP2A expression by transfecting siRNA in GLC82 and A549 cells and confirmed by qRT-PCR and Western blot (Fig. 3A). To characterize the proliferation ability of TOP2A in LUAD, we performed the colony-formation assay in GLC82 and A549 cells. As shown in Figure 3B, the colonies were markedly reduced in both TOP2A siRNA treated cell lines compared with control. The CCK8 assay also indicated that GLC82 and A549 cells with TOP2A siRNA transfection decreased growth (Fig. 3C), which was consistent with the colony-formation assay.

Lung cancer is characterized by its invasion hence we investigate whether TOP2A knockdown influences lung adenocarcinoma invasion and migration. The transwell invasion assay was performed to determine the invasion ability of GLC82 and A549 cells. The result indicated that the number of cells with transfected TOP2A siRNA was significantly reduced as compared with control (Fig. 3D). To further investigate the effect of TOP2A knockdown on migration, wound healing assay was performed and it revealed a significant attenuation of migration in GLC82 and A549 cells with TOP2A siRNA compared to control (Fig. 3E). Collectively, these results showed that TOP2A is a crucial regulator of cell proliferation, invasion and migration.

### Inhibition TOP2A has an impact of p53 pathway related gene expression

**Figure 4:**
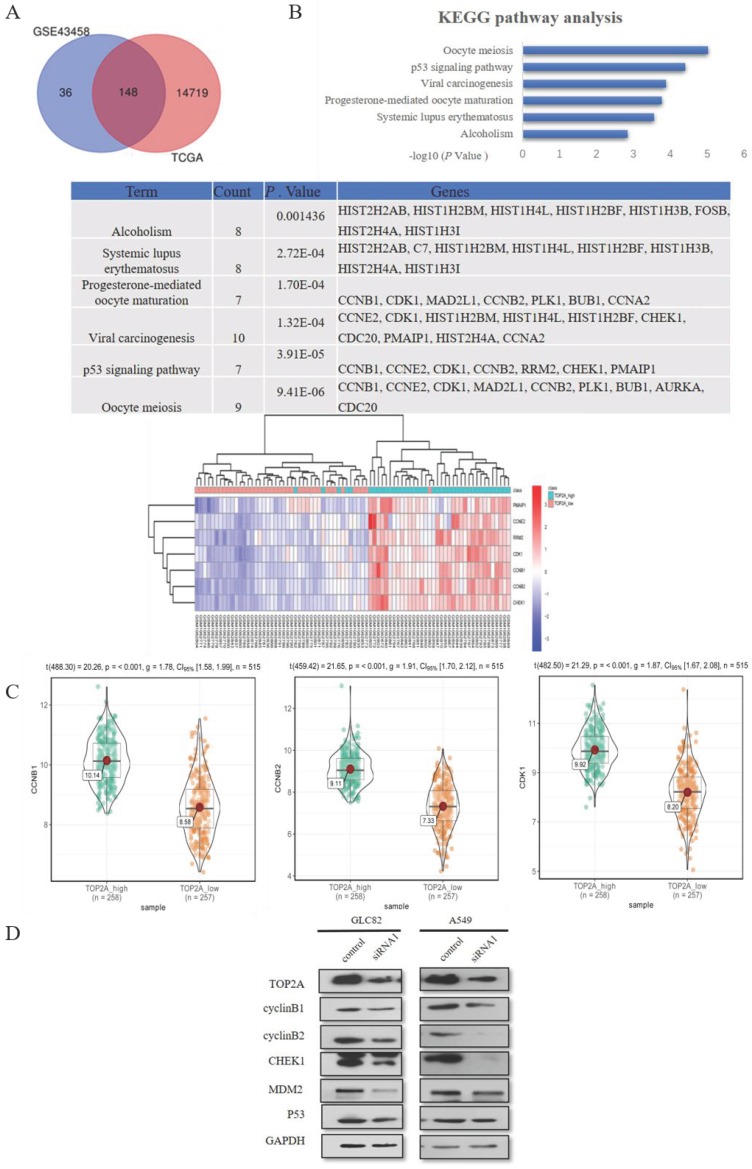
TOP2A is involved in p53 pathway. A. By analyzing GEO and TCGA databases, 148 mRNAs were found in TOP2A high and low groups. KEGG pathway enrichment analysis showed the top 3 most enriched pathways were Oocyte meiosis, p53 signaling pathway and Viral carcinogenesis, respectively. B. The heat map indicated significantly differentially expressed genes of P53 pathway between TOP2A-high and TOP2A-low group. CCNB1, CCNE2, CDK1, CCNB2, RRM2, CHEK1 and PMAIP1 were significantly upregulated in TOP2A high expression group. C. The expression levels of CCNB1, CCNB2 and CHEK1 were further identified by the violet plots, which demonstrated these genes expression levels were positively related to TOP2A. D. The expression levels of p53 related genes (cyclinB1 (CCNB1), cyclinB2 (CCNB2), CHEk1, MDM2, p53) in GLC82 and A549 cells with transfected TOP2A siRNA or control were identified by Western blot. Downregulation of the expression levels of CCNB1, CCNB2 and CHEk1 was observed in lung adenocarcinoma cells with siRNA compared with control. There was no difference in the expression levels of p53 and MDM2 between siRNA and control groups. These results indicated that TOP2A regulates the expression levels of p53 pathway related genes.

To explore the mechanism how the knockdown of TOP2A inhibits the proliferation and motility ability of GLC82 and A549 cells, we used GSE43458 to identify 184 differentially expressed mRNAs between TOP2A high and low group (Fig. 4A). We performed the same analysis from TCGA to find 14867 differentially expressed mRNAs. In total, 148 common differentially expressed mRNAs were seeked out in both GSE43458 and TCGA. Subsequently these mRNAs were taken into DAVID online database for enrichment analysis and we found 7 significantly pathways: oocyte meiosis, p53 signaling pathway, viral carcinogenesis, progesterone-mediated oocyte maturation, systemic lupus erythematosus and alcoholism. The association between TOP2A and p53 signaling pathway is unknown and p53 pathway participates in the regulation of many types of cancer, therefore we investigated related genes involving p53 pathway between TOP2A high and low groups. CyclinB1 (CCNB1), CCNE2, CDK1, cyclinB2 (CCNB2), RRM2, CHEK1 and PMAIP1 were significantly upregulated in TOP2A high expression group (Fig. 4B). As shown in Fig. 4C, the violin plots demonstrated the median, p value and 95% CI of CCNB1, CCNB2 and CHEK1 between high and low TOP2A expression group in TCGA. CCNE2, CDK1, RRM2 and PMAIP1 were shown in Fig. S1C. These data showed the expression levels of CCNB1, CCNB2, CHEK1, CCNE2, CDK1, RRM2 and PMAIP1 in TCGA were positively associated with the expression of TOP2A, which consistent with the heat map (GSE43458).

Some studies reported TOP2A regulates cell cycle and peaks in the G2/M phase 20, so we selected CCNB1 and CCNB2 affecting G2/M phase in p53 signaling pathway as downstream to confirm TCGA and GEO dataset analysis. Also, we examined p53 related genes (p53, MDM2 and CHEK1) alteration in lung adenocarcinoma cells after TOP2A siRNA or control transfection (Fig. 4D). TOP2A siRNA cells significantly decreased CCNB1, CCNB2 and CHEK1 expression. Gently downregulation of MDM2 was also measured in TOP2A siRNA cells, but there was no striking difference of MDM2 and p53 in siRNA cells in contrast to control cells. These results showed that TOP2A is correlated to p53 pathway related gene and TOP2A influences cell proliferation and metastasis through modulating CCNB1 and CCNB2.

## Discussion

TOP2A is expressed in rapidly dividing cells, mainly modulating DNA structure. And accumulating reports revealed that TOP2A is overexpressed in many types of tumors 9-12. Furthermore, TOP2A was a recently identified target of anti-cancer drugs, such as Anthracyclines (approved for the treatment of breast cancer) and Etoposide (approved for treating small-cell lung cancer, ovarian and testicular, choriocarcinoma, lymphoma, and acute myeloid leukemia) 4, 21-25. Recent studies have revealed that topoisomerase II inhibitors regulate cancer cell proliferation by involving in metabolism, apoptosis and JAK2-STAT1-CXCL1 pathway 26-29. In terms of metabolism, a study reported that topoisomerase inhibitors can directly induce defects in nucleic acid metabolisms, which has become a potential lethal 30. However, the biological function of TOP2A in lung adenocarcinoma was far from being thoroughly understood.

Initially, we evaluated the expression of TOP2A in lung adenocarcinoma. Our results showed that high TOP2A expression in lung adenocarcinoma, and the expression level of TOP2A was inversely associated with the prognosis of patients with LUAD. High expression of TOP2A has a worse prognosis in terms of overall survival and recurrence-free survival. As indicated in previous studies, our results confirmed that the overexpression of TOP2A in cancer is related to worse prognosis 13, 14, 27-29. TOP2A has been suggested that it is involved in tumorigenesis and development and its inhibitors can effectively kill cancer cells 26, 30. A study based on Oncomine database was performed to compare the overall survival of non-small-cell lung cancer among different topoisomerase isoforms. The result shows that TOP2A significantly upregulates in LUAD than other isoforms and is associated with poor prognosis 31. These findings revealed that TOP2A could be identified as a significant prognostic factor for lung adenocarcinoma patients.

TOP2A has been shown to be a prognostic indicator for inhibiting the proliferation and invasion of pancreatic cancer and bladder cancer. In this regard, we further investigated the role of TOP2A in GLC82 and A549 and found that TOP2A downregulation inhibited cells' proliferation and motility. To gain insights into how TOP2A induced proliferation and invasion in cancer cells, we then explored which signaling pathways were involved in this process. Tumor growth and metastasis are multifactorial process that is modulated by complex process and diverse pathways. Several lines of evidence show that TOP2A regulate signaling pathway in cancer. Pei et al reported that TOP2A, working as a co-activator of β-catenin, activates EMT process to promote tumor metastasis 12. In our present study, we found that p53 pathway is related to TOP2A expression through bioinformatics analysis. The significantly differentially expressed genes of P53 pathway includng CCNB1, CCNE2, CDK1, CCNB2, RRM2, CHEK1 and PMAIP1 were significantly upregulated in TOP2A-high group. Taylor et al reported that p53 overexpression results in G2 arrest and represses CHEK1 and CCNB1 32. Some literatures reported that response to TOP2A agents is influenced by several factors, but cell cycle checkpoints play a crucial role in resistance and sensitivity. Because TOP2A regulates G2 and M phase 33, 34 and p53 is involved in cell cycle, we focused on whether the expression levels of CCNB1, CCNB2 and CHEK1 upregulated in lung adenocarcinoma when knocking down TOP2A. According to the results of Western blot, we discovered that the knockdown of TOP2A results in reduction in the expression levels of CCNB1, CCNB2 and CHEK1 and no change in p53. A growing body of literature indicated that p53-MDM2 axis is associated with tumor resistant and regulates cell proliferation 35-37. Inspired by previous studies, we hypothesized that TOP2A may not participate in the regulation of p53-MDM2 axis. To test this hypothesis, we explored the effect of inhibition of TOP2A to the expression level of MDM2. These results revealed that the expression level of MDM2 has no alteration and TOP2A is not involved in p53-MDM2 axis. Accumulating studies revealed that CCNB1, CCNB2 and TOP2A are associated with the prognosis of cancer and active carcinoma pathogenesis 38-40. Consistent with these studies, our study indicated that TOP2A targeting CCNB1 and CCNB2 promote cell proliferation and motility. However, little is known about the relationship between TOP2A and CHEK1. Yao et al reported that more inhibition of cell growth was caused by the combination of Chk1 inhibitor and etoposide than etoposide alone 41. So, we speculated that TOP2A and CHEK1 may co-regulate the expression levels of CCNB1 and CCNB2. All these findings demonstrated that TOP2A targeting CCNB1 and CCNB2 promotes the growth of GLC82 and A549 cells.

To sum up, our study revealed that TOP2A was highly expressed in lung adenocarcinoma compared with matched adjacent normal tissues and high expression of TOP2A was associated with poor prognosis for LUAD patients. Moreover, knockdown of TOP2A in A549 and GLC82 cells significantly inhibited cell proliferation, migration and invasion. Inhibition of TOP2A reduced the expression levels of CCNB1 and CCNB2, which indicated that TOP2A targeting CCNB1 and CCNB2 promotes GLC82 and A549 cells proliferation and metastasis. These results showed that TOP2A is crucial in the tumorigenesis of lung adenocarcinoma. Our findings suggest that TOP2A may not only be an effectively predictive and prognostic biomarker but also be a novel rationale for targeting TOP2A in the treatment of lung adenocarcinoma. Further studies of the molecular mechanism for the TOP2A involved in signaling pathway should be explored.

## Supplementary Material

Supplementary figure and table.

## Figures and Tables

**Table 1:**
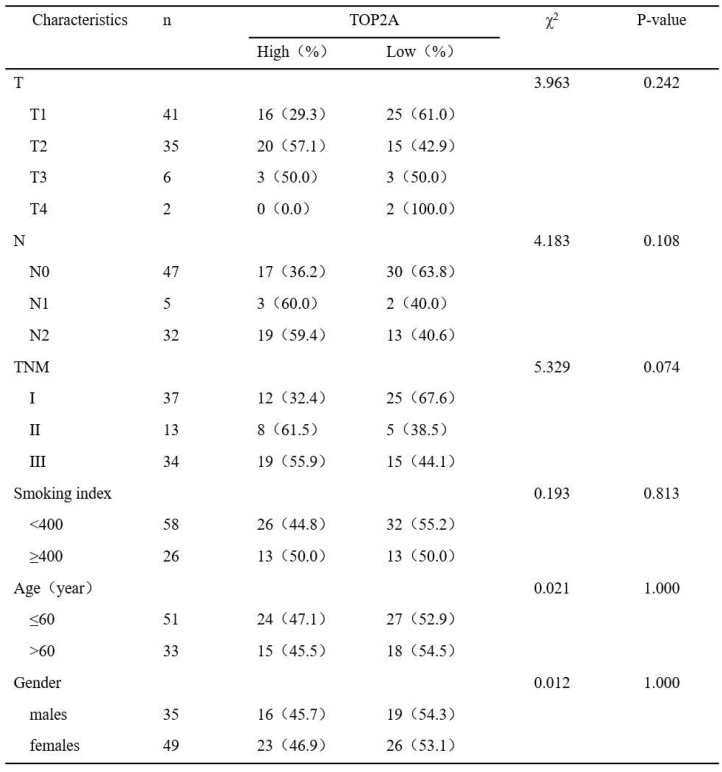
Correlation between TOP2A level and clinicopathologic characteristics of LUAD patients (p<0.05 statistically significant)

**Table 2:**
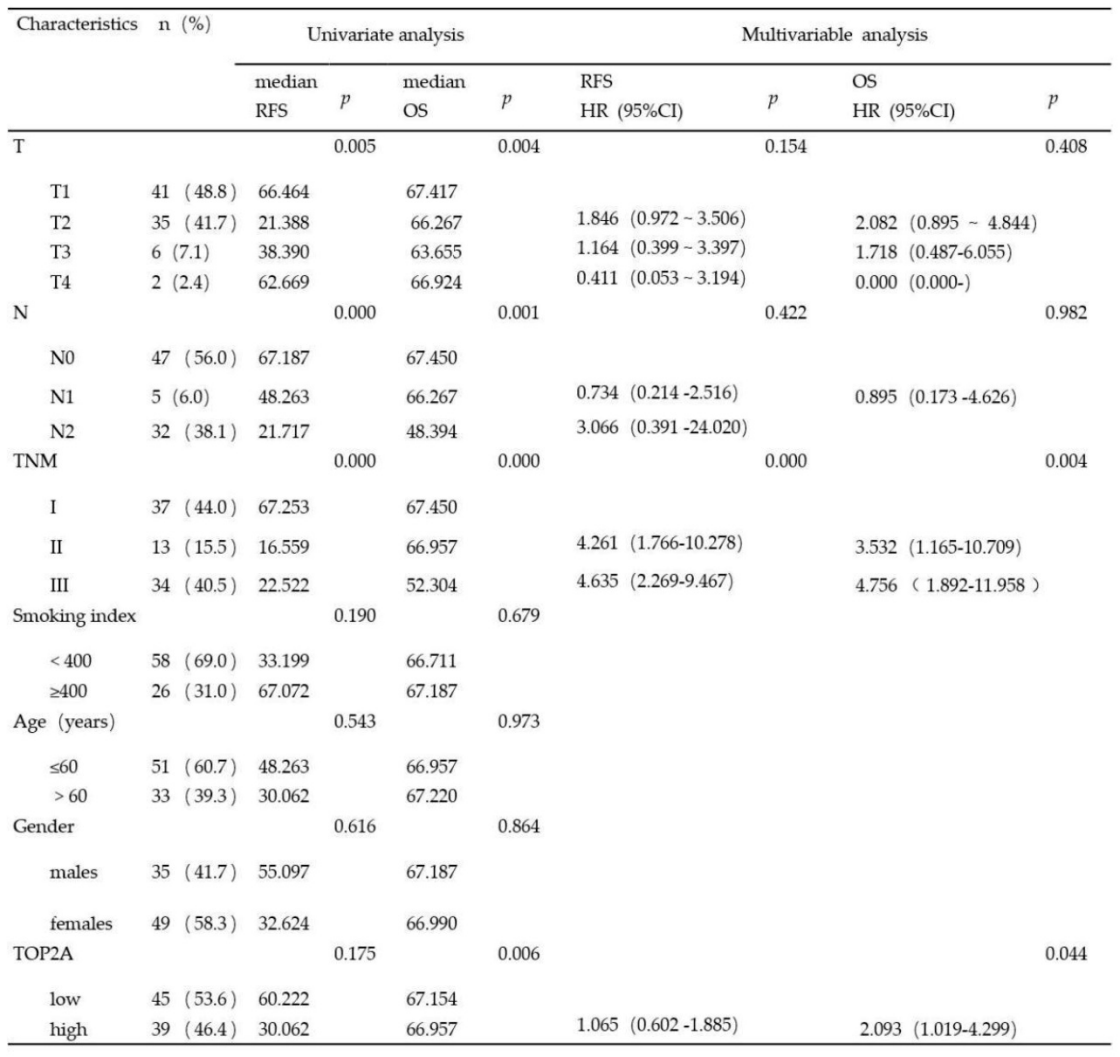
Univariate and multivariate analysis of the relationship between clinical and survival in patients with lung adenocarcinoma (p<0.05 statistically significant)

## References

[1] Siegel RL, Miller KD, Jemal A (2018). Cancer statistics, 2018.

[2] Wang J, Zhou Y, Ma L, Cao S, Gao W, Xiong Q (2019). CIAPIN1 Targeted NHE1 and ERK1/2 to Suppress NSCLC Cells' Metastasis and Predicted Good Prognosis in NSCLC Patients Receiving Pulmonectomy. Oxid Med Cell Longev.

[3] Chien C-M, Yang J-C, Wu P-H, Wu C-Y, Chen G-Y, Wu Y-C (2019). Phytochemical naphtho[1,2-b] furan-4,5-dione induced topoisomerase II-mediated DNA damage response in human non-small-cell lung cancer. Phytomedicine.

[4] Hirsch FR, Scagliotti GV, Mulshine JL, Kwon R, Curran WJ, Wu Y-L (2017). Lung cancer: current therapies and new targeted treatments. Lancet.

[5] Nitiss JL (2009). DNA topoisomerase II and its growing repertoire of biological functions. Nat Rev Cancer.

[6] Vos SM, Tretter EM, Schmidt BH, Berger JM (2011). All tangled up: how cells direct, manage and exploit topoisomerase function. Nat Rev Mol Cell Biol.

[7] Wang JC (2002). Cellular roles of DNA topoisomerases: a molecular perspective. Nat Rev Mol Cell Biol.

[8] Thakurela S, Garding A, Jung J, Schübeler D, Burger L, Tiwari VK (2013). Gene regulation and priming by topoisomerase IIα in embryonic stem cells. Nat Commun.

[9] Heestand GM, Schwaederle M, Gatalica Z, Arguello D, Kurzrock R (2017). Topoisomerase expression and amplification in solid tumours: Analysis of 24,262 patients. Eur J Cancer.

[10] Hooks KB, Audoux J, Fazli H, Lesjean S, Ernault T, Dugot-Senant N (2018). New insights into diagnosis and therapeutic options for proliferative hepatoblastoma.

[11] Kolberg M, Høland M, Lind GE, Ågesen TH, Skotheim RI, Hall KS (2015). Protein expression of BIRC5, TK1, and TOP2A in malignant peripheral nerve sheath tumours-A prognostic test after surgical resection. Mol Oncol.

[12] Pei Y-F, Yin X-M, Liu X-Q (2018). TOP2A induces malignant character of pancreatic cancer through activating β-catenin signaling pathway. Biochim Biophys Acta Mol Basis Dis.

[13] Brase JC, Schmidt M, Fischbach T, Sültmann H, Bojar H, Koelbl H (2010). ERBB2 and TOP2A in breast cancer: a comprehensive analysis of gene amplification, RNA levels, and protein expression and their influence on prognosis and prediction. Clin Cancer Res.

[14] Labbé DP, Sweeney CJ, Brown M, Galbo P, Rosario S, Wadosky KM (2017). TOP2A and EZH2 Provide Early Detection of an Aggressive Prostate Cancer Subgroup. Clin Cancer Res.

[15] Zhang R, Xu J, Zhao J, Bai JH (2018). Proliferation and invasion of colon cancer cells are suppressed by knockdown of TOP2A. J Cell Biochem.

[16] Mizuno H, Kitada K, Nakai K, Sarai A (2009). PrognoScan: a new database for meta-analysis of the prognostic value of genes. BMC Med Genomics.

[17] Irizarry RA, Hobbs B, Collin F, Beazer-Barclay YD, Antonellis KJ, Scherf U (2003). Exploration, normalization, and summaries of high density oligonucleotide array probe level data. Biostatistics.

[18] Xiao Y, Feng M, Ran H, Han X, Li X (2018). Identification of key differentially expressed genes associated with non-small cell lung cancer by bioinformatics analyses. Mol Med Rep.

[19] Zhang L, Luo B, Dang Y-W, He R-Q, Peng Z-G, Chen G (2019). Clinical Significance of microRNA-196b-5p in Hepatocellular Carcinoma and its Potential Molecular Mechanism. J Cancer.

[20] Nakada S, Katsuki Y, Imoto I, Yokoyama T, Nagasawa M, Inazawa J (2006). Early G2/M checkpoint failure as a molecular mechanism underlying etoposide-induced chromosomal aberrations. J Clin Invest.

[21] Wang S, Zimmermann S, Parikh K, Mansfield AS, Adjei AA (2019). Current Diagnosis and Management of Small-Cell Lung Cancer. Mayo Clin Proc.

[22] Bailly C (2012). Contemporary challenges in the design of topoisomerase II inhibitors for cancer chemotherapy. Chem Rev.

[23] Chen SH, Chan N-L, Hsieh T-s (2013). New mechanistic and functional insights into DNA topoisomerases. Annu Rev Biochem.

[24] Nitiss JL (2009). Targeting DNA topoisomerase II in cancer chemotherapy. Nat Rev Cancer.

[25] Wu C-C, Li T-K, Farh L, Lin L-Y, Lin T-S, Yu Y-J (2011). Structural basis of type II topoisomerase inhibition by the anticancer drug etoposide. Science.

[26] Wasim L, Chopra M (2018). Synergistic anticancer effect of panobinostat and topoisomerase inhibitors through ROS generation and intrinsic apoptotic pathway induction in cervical cancer cells. Cell Oncol (Dordr).

[27] Liu J, Qu L, Meng L, Shou C (2019). Topoisomerase inhibitors promote cancer cell motility via ROS-mediated activation of JAK2-STAT1-CXCL1 pathway. J Exp Clin Cancer Res.

[28] Taymaz-Nikerel H, Karabekmez ME, Eraslan S, Kırdar B (2018). Doxorubicin induces an extensive transcriptional and metabolic rewiring in yeast cells. Sci Rep.

[29] Demel H-R, Feuerecker B, Piontek G, Seidl C, Blechert B, Pickhard A (2015). Effects of topoisomerase inhibitors that induce DNA damage response on glucose metabolism and PI3K/Akt/mTOR signaling in multiple myeloma cells. Am J Cancer Res.

[30] Chowdhury SR, Majumder HK (2019). DNA Topoisomerases in Unicellular Pathogens: Structure, Function, and Druggability. Trends Biochem Sci.

[31] de Resende MF, Vieira S, Chinen LTD, Chiappelli F, da Fonseca FP, Guimarães GC (2013). Prognostication of prostate cancer based on TOP2A protein and gene assessment: TOP2A in prostate cancer. J Transl Med.

[32] D Arcy N, Gabrielli B (2017). Topoisomerase II Inhibitors and Poisons, and the Influence of Cell Cycle Checkpoints. Curr Med Chem.

[33] Broderick R, Nieminuszczy J, Blackford AN, Winczura A, Niedzwiedz W (2015). TOPBP1 recruits TOP2A to ultra-fine anaphase bridges to aid in their resolution. Nat Commun.

[34] Park J-E, Yi H, Kim Y, Chang H, Kim VN (2016). Regulation of Poly(A) Tail and Translation during the Somatic Cell Cycle. Mol Cell.

[35] Hainsworth JD, Greco FA (1995). Etoposide: twenty years later. Ann Oncol.

[36] Tretiakova M, Turkyilmaz M, Grushko T, Kocherginsky M, Rubin C, Teh B (2006). Topoisomerase IIalpha in Wilms' tumour: gene alterations and immunoexpression. J Clin Pathol.

[37] Zaczek AJ, Markiewicz A, Seroczynska B, Skokowski J, Jaskiewicz J, Pienkowski T (2012). Prognostic significance of TOP2A gene dosage in HER-2-negative breast cancer. Oncologist.

[38] Nag S, Zhang X, Srivenugopal KS, Wang MH, Wang W, Zhang R (2014). Targeting MDM2-p53 interaction for cancer therapy: are we there yet?. Curr Med Chem.

[39] Hou G-X, Liu P, Yang J, Wen S (2017). Mining expression and prognosis of topoisomerase isoforms in non-small-cell lung cancer by using Oncomine and Kaplan-Meier plotter. PLoS ONE.

[40] Minotti G, Menna P, Salvatorelli E, Cairo G, Gianni L (2004). Anthracyclines: molecular advances and pharmacologic developments in antitumor activity and cardiotoxicity. Pharmacol Rev.

[41] Nomura K, Klejnot M, Kowalczyk D, Hock AK, Sibbet GJ, Vousden KH (2017). Structural analysis of MDM2 RING separates degradation from regulation of p53 transcription activity. Nat Struct Mol Biol.

