# Supplementary material for: TOP2A Promotes Lung Adenocarcinoma Cells' Malignant Progression and Predicts Poor Prognosis in Lung Adenocarcinoma
Source: J Cancer. 2020 Feb 10;11(9):2496–508. doi: 10.7150/jca.41415 (PMC7066024; doi:10.7150/jca.41415)
Supplement: Supplementary file 1 — Supplementary figure and table. [file jcav11p2496s1.pdf]

Supplementary Materials:

Fig S1A

The constructed PPI network of the common DEGs screened out from the four datasets.

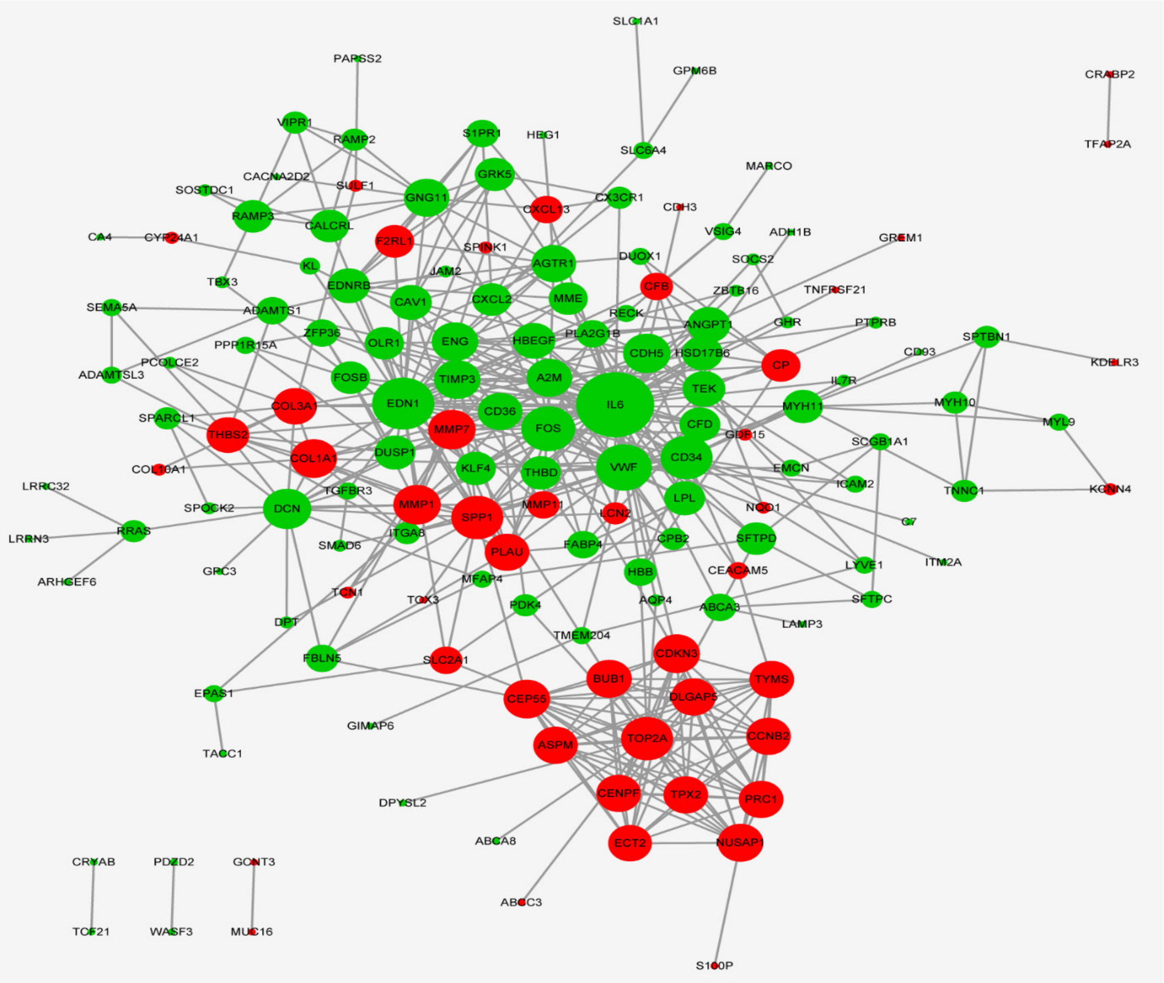

The PPI network was constructed by 154 DEGs filtered out from STRING database. The red and green nodes represent up- and down-regulated DEGs, respectively. And the larger the node, the higher the degree.

Table S1

The connectivity degrees corresponding to hub genes in the PPI network

Gene	Degree	Gene	Degree
IL6	48	BUBI	13

EDN1	31	NUSAP1	13
VWF	24	A2M	13
FOS	22	GNG11	13
SPP1	20	CENPF	12
TOP2A	20	PLAU	12
CD34	20	CD36	12
TIMP1	18	PRC1	12
DCN	16	TPX2	12
MMP1	15	CCNB2	12
MMP7	15	ASPM	12
CDH5	15	AGTR1	12
CEP55	14	TYMS	12
TIMP3	14	DLGAP5	12
CDKN3	14	COL3A1	11
ENG	14	ECT2	11
COL1A1	14		

Fig S1B

The Kaplan-Meier curves of the remaining 18 hub up-regulated genes obtained by R software

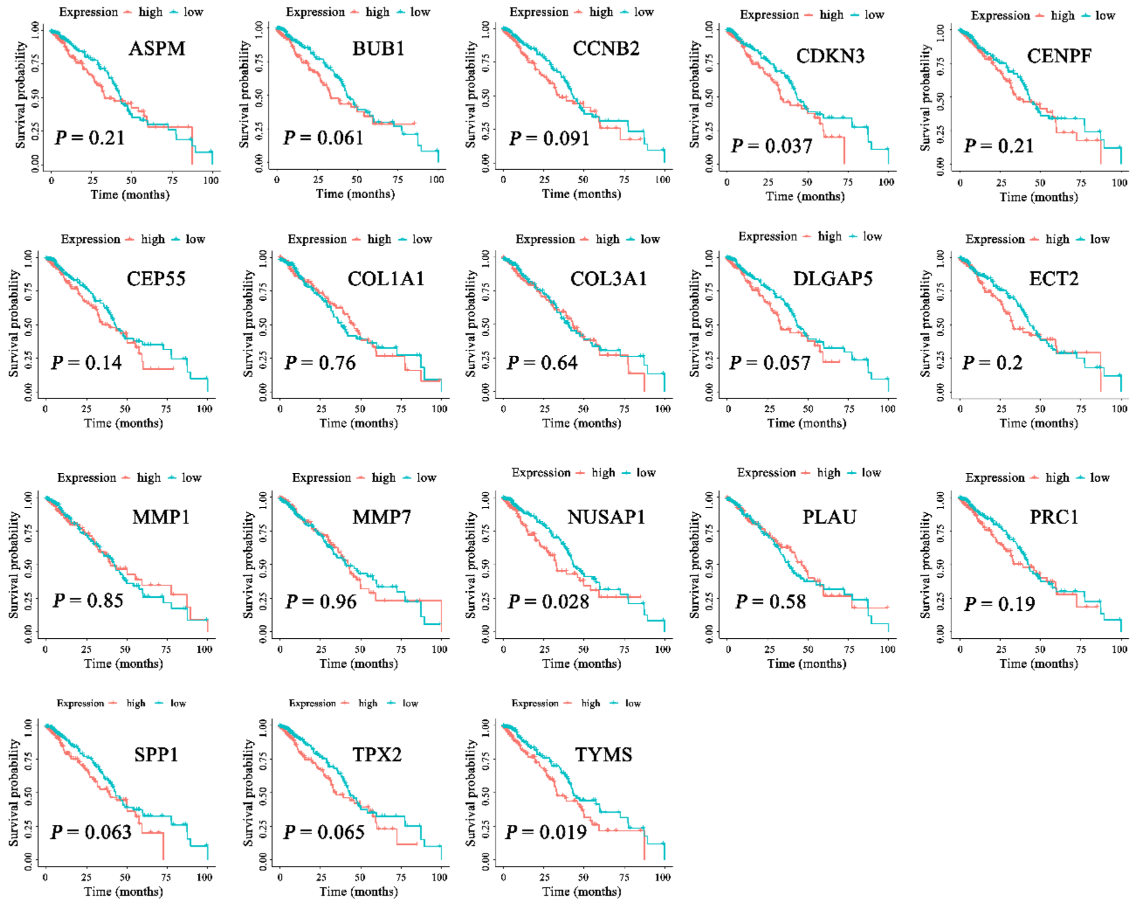

The Kaplan-Meier curves demonstrated that the expression levels of these genes were not significantly related to the prognosis of patients （all $P > 0.01$）

Fig S1C

The violin plots demonstrated the median, p value and 95% CI of CCNE2, CDK1, RRM2 and

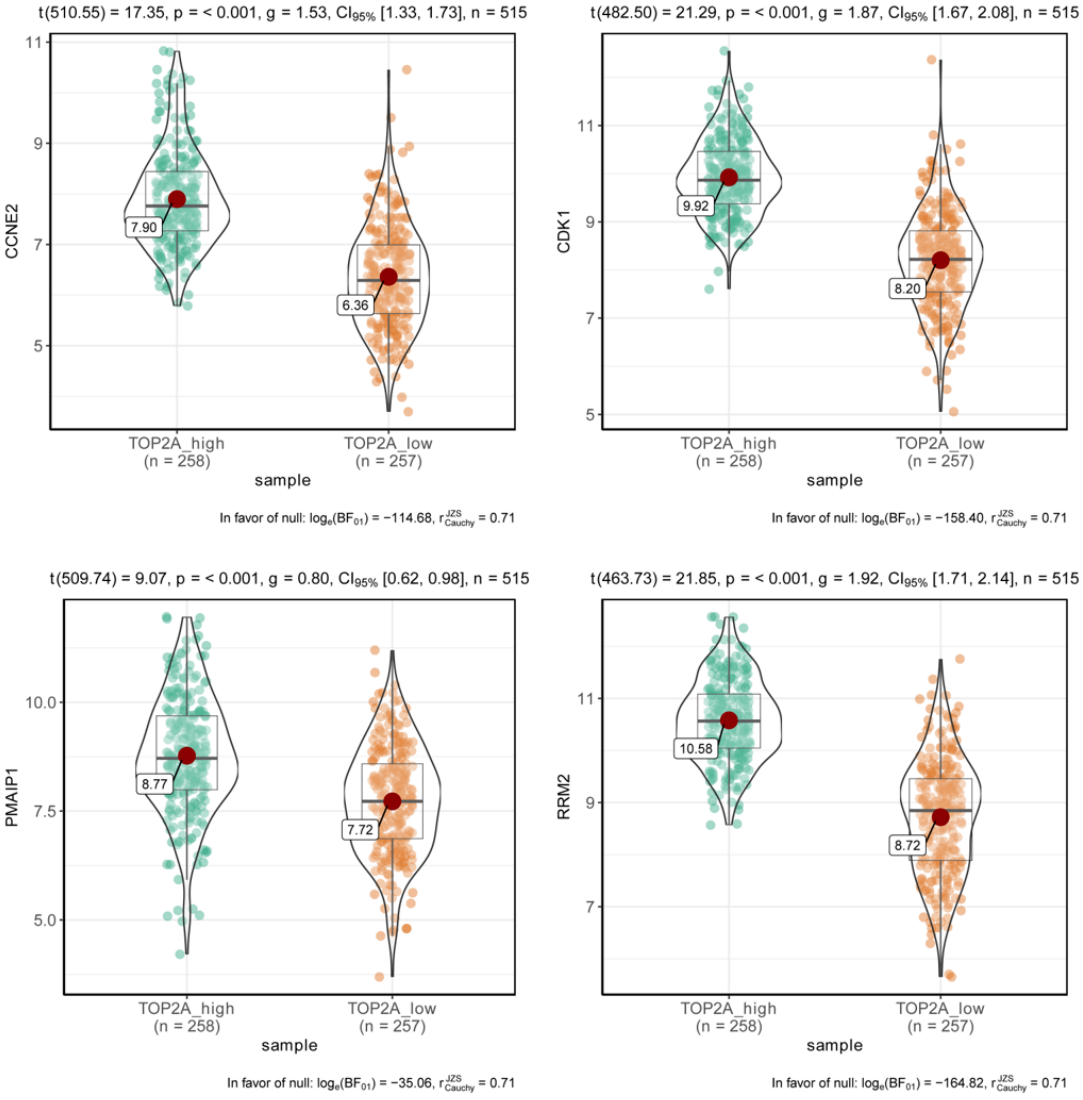

PMAIP1 between high and low TOP2A expression group in TCGA.
